# Muscle-clipping suturing for closure of a gastric defect after endoscopic submucosal dissection for a type 3 neuroendocrine neoplasm

**DOI:** 10.1055/a-2868-7987

**Published:** 2026-05-21

**Authors:** Felipe Ramos-Zabala, Alejandra Alzina-Pérez, Edwin Mejía, Marian García-Mayor, Raúl José Díaz-Molina, Luis Moreno-Almazán

**Affiliations:** 1 Servicio de Gastroenterología 181073 Hospital Universitario HM MontepríncipeMadridSpain; 2 Servicio de GastroenterologíaHospital Universitario HM Puerta del SurMadridSpain; 3 Centro Universitario HM Hospitales de Ciencias de la Salud (CUHMED), Universidad Camilo José CelaMadridSpain; 4 Universidad San Pablo-CEUCEU UniversitiesMadridSpain; 5 Servicio de Anatomía PatológicaHospital Universitario HM Puerta del SurMadridSpain

Selected type 3 gastric neuroendocrine neoplasms (g-NENs) <20 mm, well differentiated and confined to the mucosa–submucosa without nodal or distant metastases, may be managed by endoscopic submucosal dissection (ESD 1 ). However, closure of large post-ESD defects in the gastric fundus remains technically demanding, as deep submucosal dead space may persist even after conventional clip approximation.

A 48-year-old woman was referred for iron-deficiency anemia. Upper gastrointestinal endoscopy revealed a 12-mm type 3 g-NEN on the anterior wall at the body–fundus junction. Endoscopic ultrasound demonstrated a hypoechoic lesion confined to the first three layers, without muscularis propria invasion. En bloc ESD was performed using a waterjet-assisted hydrodissection system and a T-type HybridKnife in probe mode (ERBEJET; Erbe, Germany 2 ). Dissection was carried out through severe submucosal fibrosis densely adherent to the muscularis propria, requiring meticulous layer identification and resulting in broad muscular exposure. R0 resection was achieved. Histology confirmed a well-differentiated, grade 1 g-NEN with negative margins.

**Fig. 1:**
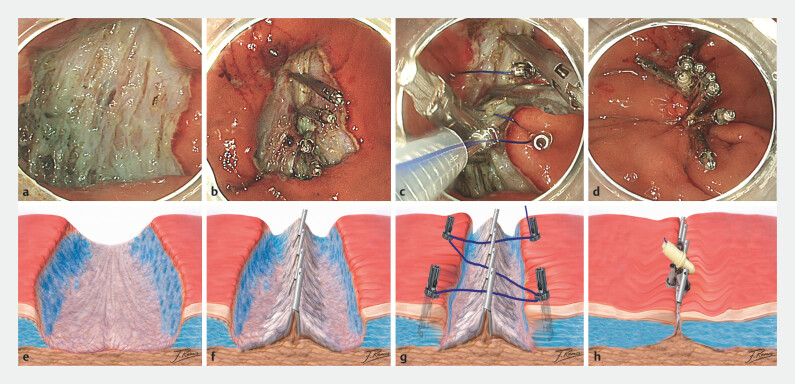
**a** Wide gastric ESD defect with exposed muscularis propria. **b** Placement of through-the-scope clips to fold the exposed muscularis propria and create a central muscle ridge. **c** Deployment of a through-the-scope tack-and-suture system (X-Tack system) anchoring the folded muscle to the surrounding deep submucosa and mucosal margins; the suture is passed between three clips along the ridge to achieve layered approximation and eliminate submucosal dead space. **d** Final multilayer closure after suture cinching and the placement of reinforcement clips. **e–h** Corresponding schematic illustrations demonstrating the same sequential steps. Illustration created by the authors (F. Ramos). ESD, endoscopic submucosal dissection.

Given the broad muscular exposure, proximal gastric location, and fibrosis-related deep defect, closure was performed using a muscle-clipping suturing strategy adapted from our origami-based colonic technique ( Video 1 , Fig. 1 ) 3 4 5 . Four Resolution Clips (Boston Scientific, USA) were applied to fold the exposed muscularis propria, creating a central muscle ridge and reducing the defect width. The folded muscle was then anchored to the surrounding deep submucosa and mucosal margins using a through-the-scope tack-and-suture system (X-Tack endoscopic HeliX tacking system; Boston Scientific, USA). The suture was deployed between three clips along the ridge, achieving progressive tissue approximation with reduction of the submucosal dead space. Two additional muscle-to-mucosa clips and one mucosa-to-mucosa clip reinforced the closure.

Origami-based muscle-clipping suturing for closure of a gastric ESD defect, demonstrating muscle folding, tack-and-suture reinforcement, and elimination of submucosal dead space. ESD, endoscopic submucosal dissection.Video 1

The patient resumed oral intake the following day and was discharged after 24 hours without adverse events. At 12 months of follow-up, no recurrence or metastasis was detected. Muscle-clipping suturing may represent a feasible and effective closure option for selected high-risk gastric ESD defects.

Endoscopy_UCTN_Code_TTT_1AO_2AO
